# A comprehensive psychological tendency prediction model for pregnant women based on questionnaires

**DOI:** 10.1038/s41598-022-26977-3

**Published:** 2023-01-02

**Authors:** Xiaosong Han, Mengchen Cao, Junru He, Dong Xu, Yanchun Liang, Xiaoduo Lang, Renchu Guan

**Affiliations:** 1 Key Laboratory for Symbol Computation and Knowledge Engineering of National Education Ministry, College of Computer Science and Technology, Jilin University, Changchun, 130012 China; 2 Department of Electrical Engineering and Computer Science and Christopher S. Bond Life Sciences Center, University of Missouri, Columbia, MO 65211 USA; 3 Zhuhai Laboratory of Key Laboratory for Symbol Computation and Knowledge Engineering of Ministry of Education, Zhuhai College of Science and Technology, Zhuhai, 519041 China; 4 Jilin Provincial Institute of Population Science and Technology, Changchun, 130000 China

**Keywords:** Classification and taxonomy, Anxiety, Data mining

## Abstract

More and more people are under high pressure in modern society, leading to growing mental disorders, such as antenatal depression for pregnant women. Antenatal depression can affect pregnant woman’s physical and psychological health and child outcomes, and cause postpartum depression. Therefore, it is essential to detect the antenatal depression of pregnant women early. This study aims to predict pregnant women’s antenatal depression and identify factors that may lead to antenatal depression. First, a questionnaire was designed, based on the daily life of pregnant women. The survey was conducted on pregnant women in a hospital, where 5666 pregnant women participated. As the collected data is unbalanced and has high dimensions, we developed a one-class classifier named Stacked Auto Encoder Support Vector Data Description (SAE-SVDD) to distinguish depressed pregnant women from normal ones. To validate the method, SAE-SVDD was firstly applied on three benchmark datasets. The results showed that SAE-SVDD was effective, with its F-scores better than other popular classifiers. For the antenatal depression problem, the F-score of SAE- SVDD was higher than 0.87, demonstrating that the questionnaire is informative and the classification method is successful. Then, by an improved Term Frequency-Inverse Document Frequency (TF-IDF) analysis, the critical factors of antenatal depression were identified as work stress, marital status, husband support, passive smoking, and alcohol consumption. With its generalizability, SAE-SVDD can be applied to analyze other questionnaires.

## Introduction

Nowadays, more and more people suffer from high pressure, which can cause some mental disorders. It is estimated that 10–30% of pregnant women are affected by antenatal depression^1–5^. Antenatal depression is a pervasive disorder with severe implications on maternal and child outcomes^6,7^. More and more clinical evidence shows that antenatal depression is one of the strongest predictors of postpartum depression^8^. It has a physical and psychological impact on women during pregnancy, which can cause anorexia, violence, drug or alcohol abuse, and adverse effects on maternal and child relationships, as well as on children’s growth environment and behavioral development. Therefore, it is important to identify pregnant women with antenatal depression. Once pregnant women are identified with a potential depression tendency early, doctors can formulate treatment strategies in time. One of the most commonly used methods is to conduct a questionnaire to collect pregnant women’s daily psychological activities, and then analyze their mental health. The most popular questionnaires include the Diagnostic and Statistical Manual of Mental Disorders DSM-IV^9^, Edinburgh Postnatal Depression Scale (EPDS)^10^, Clinical Interview Schedule-Revised (CIS-R)^11^, the Beck Depression Inventory^12^, the Mini-International Neuro-psychiatric Interview-Plus (MINI)^13^, and Patient Health Questionnaire-8 (PHQ-8)^14^.

At present, antenatal depression is mainly judged by questionnaires. Cheng et al.^15^ used descriptive statistics, Pearson and Spearman’s correlation to analyze the data collected by questionnaires. Zhang et al.^16^ collected data through questionnaire based on self-rating depressions cale(SDS)^17^, and used multiple logistic regression analysis and tendency score matching to predict depression. But questionnaires are limited and cannot reflect the causal factors of the disorder well. Therefore, this paper designed and applied a novel questionnaire in an antenatal survey of pregnant women. Then collected data was used to predict whether the pregnant woman was in severe antenatal depression. Compared with previous questionnaires, our questionnaire is based on the pregnant women’s daily life, designed with more details and easier to discover the prevalence and determinants of depression. Furthermore, we organized these questions so that we could analyze the disease from different aspects. The samples with antenatal depression account for only a small portion, while most of them are healthy. Besides, each sample contains up to 147 features. Hence, we were faced with an imbalanced and high-dimensional classification problem. To address this problem, we applied a one-class model for the single classification problem. Such a strategy has been widely used in network traffic anomaly detection, fault diagnosis, credit card fraud detection and other fields^18–20^.

In this study, we applied Support Vector Data Description (SVDD), which describes the boundary of only one class samples to distinguish target data^21^. It is suitable for solving the single classification problem with high dimensional and limited sample data^22^. However, when the target data is unevenly distributed, and the density of the data varies greatly, the classification performance of SVDD is negatively affected. To address this problem, Li and Manevitz^23,24^ considered that since the classification boundary of the classifier is determined by a small number of non-zero support vectors, the potential support vector samples are selected and used as a training set to construct the classification boundary, thereby improving the classifier training speed by reducing the training set. The final classification performance depends on the scale of the training set and the set’s parameters. Zhang^25^ introduced the weighted and dynamic inertia factor based on the original simulated annealing method and particle swarm optimization algorithm to improve the parameter optimization process. The optimization strategy also effectively improves the classification performance on the traffic classification problem. To improve the training efficiency of the classifier, Xu and Chen^26,27^ introduced the idea of parallel learning to a single classification problem. The training set is divided into K subclasses by K-means clustering, and then each subclass is trained by SVDD. The strategy also effectively improves the classification accuracy on the problems with a large-scale, large noise and low population density dataset. However, the K value is difficult to set. Cano^28^ proposed a Pareto-based multi-objective genetic algorithm for feature extraction and data visualization. The algorithm is designed to converse balanced and unbalanced data to achieve high classification and visualization performance, outperforming existing feature extraction algorithms. Guan^29^ proposed to generate feature vector space in a feature selection module and feature vectors are used to train softmax regressor, and completed the task of recommending journals. Krawczyk^30^ believed that, reducing the training set could reduce the classification time and classifier complexity while filtering out internal noises and simplifying the data description boundaries. Two approaches were proposed to achieve this goal. The first one is a flexible framework that adjusts any instance reduction method to one-class of scenarios by introducing significant artificial outliers. The second one is a novel modification of the evolutionary instance reduction technique based on differential evolution and uses consistency measures for model evaluation in filter or wrapper modes. It is a powerful native one-class solution that does not require access to counterexamples. Both of the algorithms can be applied to any single-class classifier. Extensive computational experiments show that the proposed methods are highly efficient techniques to reduce complexity and improve classification performance in single class scenarios. Wu et al.^31^ combined Affinity Propagation (AP) clustering algorithm and SVDD, and used an improved Particle Swarm Optimization (PSO) algorithm to evolve the parameters of SVDD.

We improved the SVDD algorithm with the idea of “divide and conquer” and proposed a Self-Adaptive SVDD (SA-SVDD)^32^ consisted of SVDD, AP clustering algorithm^33^ and Particle Swarm Optimization (PSO)^34^. The experimental results showed that the performance of SA-SVDD was significantly improved compared to some classic single classification algorithms. However, when the data is unbalanced, and the feature is high-dimensional and spare, SA-SVDD does not perform well. So the Stacked Automatic Encoder (SAE)^35^ was used to reduce the data dimension in this study, then SAE-SVDD combining SAE and SA-SVDD was proposed. The main flow of the SAE-SVDD is to conduct SAE firstly to embed the data into a space of lower dimension. SA-SVDD can classify the reduced-dimensional data. The experimental results on the LIBSVM datasets demonstrated that SAE-SVDD had a significant improvement on classification performance and running time over classical single classification algorithms, including SVDD, SA-SVDD, and four deep learning-based models. Then, SAE-SVDD was applied to the antenatal depression classification problem and achieved better performance.

This paper is organized as follows. “Introduction” briefly introduces the research background. SA-SVDD and SAE are presented in “Literature review”. In “Methods”, the proposed SAE-SVDD is described, and three benchmark data sets are employed to verify this method. In “Experiments on antenatal depression dataset questionnaire”, SAE-SVDD is applied to distinguish pregnant women with antenatal depression. Finally, we summarize and discuss the research results in “Discussion” and “Conclusion”.

## Literature review

### Self-adaptive support vector data description

Based on the imbalanced data, the one-class classifier can only be trained for a type of data. However, the data distribution is often diverse. If only one hypersphere is used to describe the sample set, the decision boundaries are inevitably not compact, resulting in reduced classification performance^36^. We have proposed an algorithm named Self-Adaptive Support Vector Data Description (SA-SVDD) to solve this problem^32^. Firstly, the training set is divided into K sub-clusters by Affinity Propagation(AP) clustering with the sample similarity. Therefore, the boundaries of each cluster are relatively compact. Secondly, the decision boundary of each cluster is described by SVDD. Finally, SVDD is used to partition the entire training set into K sub-hyperspheres. One only needs to judge whether a new sample belongs to one of the hyperspheres to predict its category. An extended PSO, Global Prediction-Based Adaptive Mutation Particle Swarm Optimization(GPAM-PSO)^37^, is used to adaptively adjust the parameters of SVDD for all the sub-clusters to improve the accuracy. The workflow of SA-SVDD is described as following.

**Table:** 

Workflow of SA-SVDD	
1.**Initialization:** the dataset;	
2.**Preference value:** Silhouette indicator is calculated to set **Preference** value of AP clustering;	
3.**Partition:** Run AP clustering on the training set to obtain K subclasses;	
4.**Parameter Optimization:** GPAM-PSO is employed to train SVDD for each subclass obtained above, and the F score of the 5-fold cross-validation is used as the fitness function;	
5.**Discriminating Boundary:** the hyperspheres of K subclasses;	
6.**Prediction:** a new sample is assessed whether it belongs to one of the K subclasses. If it belongs to one of them, it is a target class sample. Otherwise, it is an abnormal class sample.	

Preference (*P*) is an essential parameter in AP clustering. We use the Silhouette indicator as the evaluation criterion to select the *P* value. The silhouette is an internal validity indicator, which applies to where the dataset category is unknown. It embodies the intra-class tightness and inter-class separation of the cluster structure as defined by Eq. (1)

1 $$\begin{aligned} s(i)=(b(i)-a(i))/{max\{a(i),b(i)\}}, \end{aligned}$$

where *a*(*i*) represents the degree of difference between point *i* and the current category, and *b*(*i*) is the minimal difference between point *i* and other categories. It is known by Eq. (1) that $-1\le s(i)\le 1$. If *s*(*i*) is expected to close to 1, it needs $a(i)\ll b(i)$. The average Silhouette value of all samples can be used to evaluate the clustering quality^38^.

The SVDD model can be described as: in a given training set $\{ x_i |x_i\in R^n \}$, a minimal hypersphere, containing as many target data points as possible, is built in the mapped high-dimensional feature space. The problem is specified as an optimization problem, and the objective functions are as Formulas (2) and (3). The goal is to minimize Formula 2.

2 $$\begin{aligned} f(R,a)=R^2+C\Sigma _i\xi _i \end{aligned}$$

s.t.:

3 $$\begin{aligned} {\left| \left| x_i-a\right| \right| }^2\le R^2+\xi _i, \xi _i \ge a, 1\le i \le N \end{aligned}$$

where the hypersphere center is *a*, and the radius is *R*; *N* is the number of *x*; $\xi _i$ is the slack variable to tolerate the outliers and relax the inequality constraints, which provides the error estimation of the decision boundary at the outlier. *C* is a specified constant as a penalty variable, which could suppress the loss brought by outliers. It can be known that the larger the value of *C*, the fewer outliers are discarded.

Combining Eqs. (3) and (2), the Lagrangian function is constructed as Eq. (4):

4 $$\begin{aligned} L=R^2+C\Sigma _i\xi _i-\Sigma _i\alpha _i\{R^2+\xi _i-({\left| \left| x_i-a\right| \right| }^2)\}-\Sigma _i\gamma _i\xi _i \end{aligned}$$

where the Lagrangian multiplier $\alpha _i\ge 0,\gamma _i\ge 0$. The partial derivatives of *L* to *R*, a and $\xi _i$ are calculated, respectively. Let these derivatives equal to 0, and the following conclusion can be achieved:

5 $$\begin{aligned} \Sigma _i\alpha _i=1, a=\Sigma _i\alpha _i x_i,0\le a_i\le C \end{aligned}$$

Substitute Eq. (5) into Eq. (4), the dual formula for the optimization problem is as follows.

6 $$\begin{aligned} \max \Sigma _i\alpha _i\phi (x_i,x_i)-\Sigma _{i,j}\alpha _i a_j \phi (x_i,x_j) \end{aligned}$$

s.t.:

7 $$\begin{aligned} \Sigma _i\alpha _i=1,0\le \alpha _i\le 1 \end{aligned}$$

where $\phi (x_i,x_i)$ represents a kernel function that maps sample points from the original space to the high-dimensional feature space; $0\le \alpha _i\le C$ indicates that the sample point is on the hypersphere plane of the classifier, called the support vector; $\alpha _i=0$ suggests that the sample point is inside the classifier; $\alpha _i=C$ indicates that the sample point is outside the constructed hypersphere. Therefore, for a new sample *z*, it can be classified by the following discriminant function.

8 $$\begin{aligned} f(z)={\left| \left| z-a \right| \right| }^2=\phi (z,z)-2\Sigma _i \alpha _i\phi (z,x_i)+\Sigma _{i,j}\alpha _i\alpha _j\phi (x_i,x_j) \end{aligned}$$

where *f*(*z*) represents the distance from the new sample to the hypersphere center *a*, if $f(z)\le 0$, the new sample is the target class, otherwise it is abnormal.

From the above description, all the parameters involved in the SA-SVDD can be adapted according to the training set. The divide-and-conquer strategy transforms the problem of building a larger hypersphere into constructing multiple smaller hyperspheres.

### Stacked automatic encoder (SAE)

**Figure 1:**
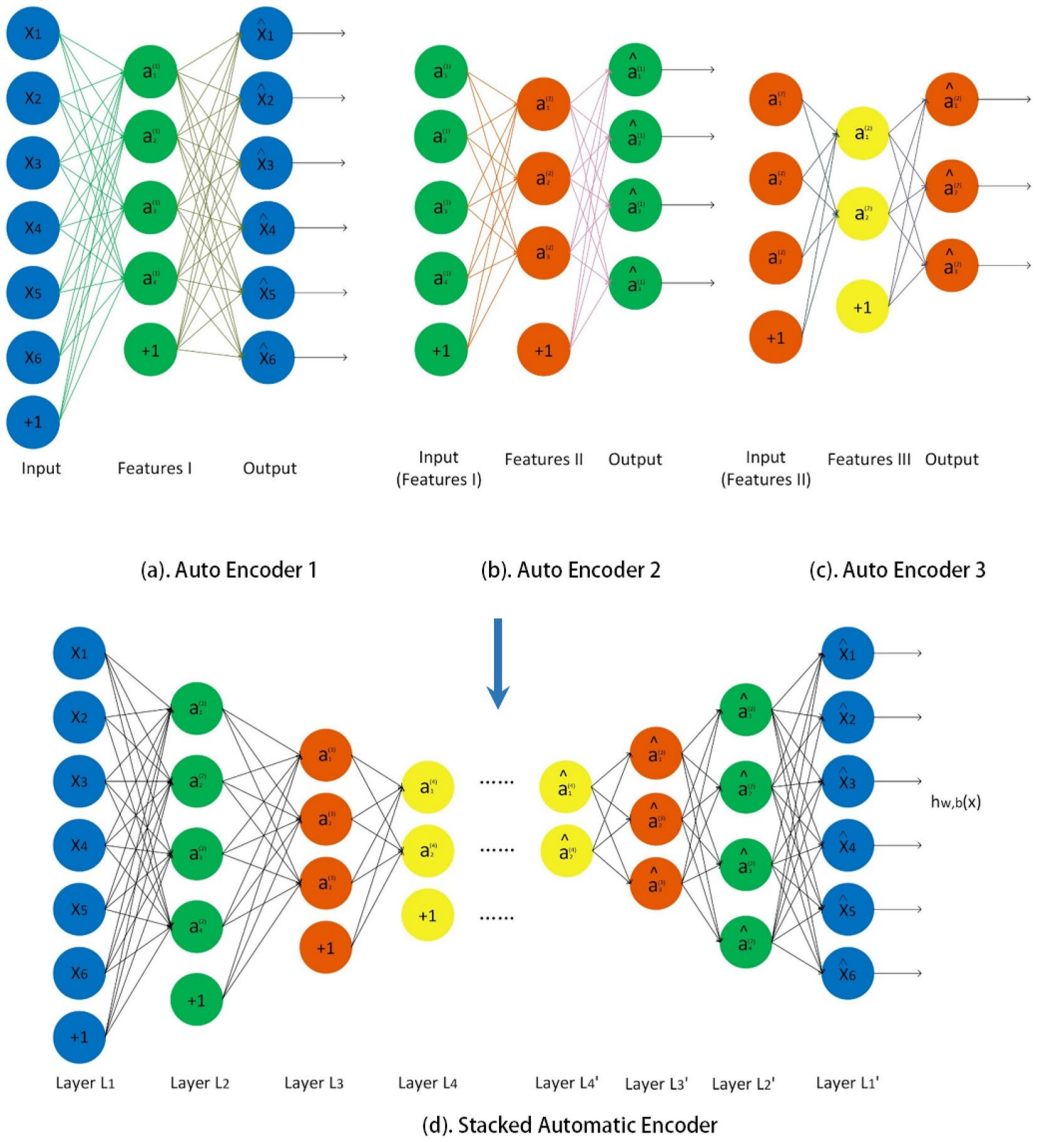
Structure of AutoEncoder.

The Automatic Encoder (AutoEncoder) is a kind of unsupervised learning neural network. The basic idea is to train the artificial neural network while the network’s output equals the input. In this way, each hidden layer is an equivalent representation of the input data. Therefore, AutoEncoder can reduce dimension and compress data^39^. The structure of AutoEncoder is shown in Fig. 1.

The Stacked AutoEncoder (SAE) is a composed neural network consisting of a group of AutoEncoders. The output of the hidden layer from the previous AutoEncoder is used as the input layer of the subsequent AutoEncoder. The layer-by-layer greedy training strategy is used to train the SAE neural network. In the encoding phase, each layer of the AutoEncoder is performed in front-to-back order.

9 $$\begin{aligned}{} & {} (a)^{(l)}=f(z^{(l)}) \end{aligned}$$

10 $$\begin{aligned}{} & {} z^{(l+1)}=W^{(l,1)}a^{(l)}+b^{(l,1)} \end{aligned}$$

In the decoding phase, each layer is performed in order from the back to the front.

11 $$\begin{aligned}{} & {} a^{(2n-l)}=f(z^{(2n-l)}) \end{aligned}$$

12 $$\begin{aligned}{} & {} z^{(2n-l+1)}=W^{(2n-l,2)}a^{(2n-l)}+b^{(2n-l,2)} \end{aligned}$$

Formulas (9) and (10) are used to calculate the hidden unit’s activation value in the encoding phase. Formula (11) and (12) are used to calculate the hidden unit’s activation value in the decoding phase. *W*(*k*, 1) and *b*(*k*, 1) represent the weights and offsets of the k$^{th}$ self-encoder. *f* represents activation function. *a* is input value. *z* is the output after the activation function. *l* means l$^{th}$ layer. Since the coupled network is symmetrical, the corresponding number of layers is $2n-l$ during the decoding phase. $a^{(n)}$ is the deepest hidden unit’s activation value, a higher-order representation of the input value.

The SAE dimension reduction process can be summarized in the following workflow.

**Table:** 

Workflow of SAE dimension reduction process	
1. Construct n-layer SAE network: the input the network structure and construct n-1 automatic coding networks	
2. Initialize SAE network parameters (activation function, learning rate, sparse target, noise ratio, etc.)	
3. Train the SAE network: input the network structure, sample data, batch number, and train each AutoEncoder sub-network obtained in the first step. (At this time, according to the characteristics of the AutoEncoder, input data is the same as the output data in the neural network training.)	

Similar to the deep neural network, the SAE also uses the layer-by-layer greedy training method. The main parameters and, activation function are set to the default values. Other parameters such as the network structure and the number of batches are determined according to the sample set’s size. Finally, the weight matrix and threshold of activation function are trained by the back-propagating method.

Take an SAE network with three hidden layers as an example to illustrate the work flow.

(1) Train the first AutoEncoder with the original input $X^{(k)}$, which can learn the first-order feature representation $a_k^{(1)}$ (green nodes) of the initial input, as shown in the left of Fig. 1a;

(2) Reuse these first-order features $a_k^{(1)}$ as input to the second self-encoder, and use them to learn the second-order feature $a_k^{(2)}$ (orange nodes), as shown in Fig. 1b;

(3) Reuse these second-order features $a_k^{(2)}$ as input to the third self-encoder, and use them to learn the third-order feature $a_k^{(3)}$ (yellow nodes), as shown in Fig. 1c;

(4) Finally, the three layers can be combined as an SAE network with multiple hidden layers, as shown in Fig. 1d.

### Text classification

Deep learning-based models for text classification^40^ are currently widely used to solve classification problem. Convolutional Neural Networks(CNNs) are mainly used for image classification and target detection in computer vision. Since Kim^38^ proposed a simple CNN-based model for text classification in 2014, more research has used CNNs for natural language processing^41^. The convolution and pooling operations of CNNs can capture local features in text, which is also useful in text classification tasks. The RNN-based model regards the text as a sequence of words, capturing the dependence between words and text structure for text classification. However, due to the vanishing/exploding gradient problem, it is difficult for general RNNs to learn long-term dependencies. LSTM^42^ can better capture long-tern dependencies by introducing a memory unit to remember values in any time interval. At the same time, the input gate, output gate and forget gate are used to adjust the process of information entering and leaving the memory unit, which effectively solves vanishing/exploding gradient in general RNNs. Later, Hinton proposed a new approach that is called Capsule Networks^43,44^. When combining the advantages of CNNs, the CapsNets can solve the problems of CNNs, such as lack of information regarding spatial relationships and misclassification based on orientation or proportion. Attention is motivated by a visual focus on different areas of the image or associated words in a sentence. In language models, attention can be interpreted as a vector of importance weights. In 2016, Zhou et al.^45^ extended the hierarchical attention model to cross-lingual emotional classification. In every language, a LSTM network was used to model documents. Then, classification was accomplished by using a layered attention mechanism, where the sentence-level attention model understood which sentences of the document were more critical for determining overall sentiment.

## Methods

### SAE-SVDD

**Figure 2:**
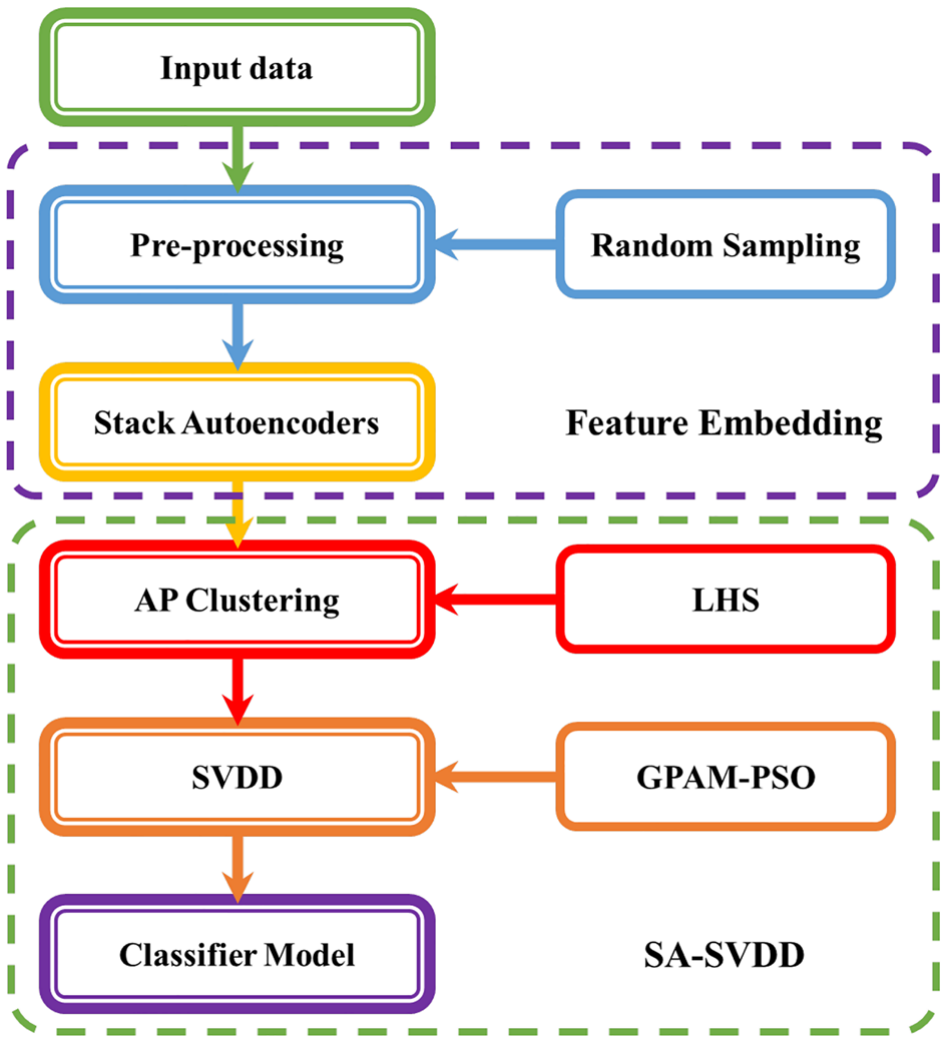
SAE-SVDD workflow.

Compared with the classic single-class classifier, SA-SVDD often achieves better performance. However, when the feature dimension increased, the time of SA-SVDD increased significantly since AP clustering and SVDD were sensitive to the feature space. SA-SVDD performed unsatisfactorily, especially for the high-dimension and sparse data. To solve this problem, SAE was combined with SA-SVDD, and the new algorithm was named Stacked Auto Encoder Support Vector Data Description (SAE-SVDD). First, SAE was used to embed the high-dimensional and sparse data into low-dimensional and dense data. Then SA-SVDD was conducted to build the classifier efficiently through the low-dimensional and dense data. The specific process of the SAE-SVDD is shown in Fig. 2. More details of the process are presented in the following workflow.

**Table:** 

Procedure of SAE-SVDD	
**Step 1.Initialization**	
1) Input the data set;	
**Step 2.Pre-processing**	
1) Remove a few irrelevant features;	
2) Divide the data set into training set and testing set randomly;	
**Step 3.Stacked Autoencoders**	
1) Build the structures of Stacked Autoencoders based on the data set;	
2) Stacked Autoencoders are trained to with the training set;	
3) Embedding feature with the encoder part;	
**Step 4.AP Clustering**	
1) Latin Hypercube Sampling (LHS) is conducted to get the candidate preference values of AP Clustering;	
2) Run AP Clustering with the candidate preference values;	
3) SIL index is calculated to evaluate the clustering result;	
4) Return the K sub-clusters with the best SIL index;	
**Step 5.SVDD**	
1) GPAM-PSO is conducted to find the parameters of SVDD of each sub-cluster,and the fitness is set as the classification results of SVDD with 5-fold cross-validation;	
2) K sub-hyperspheres are constructed by SVDD with the best parameters;	
**Step 6.Classifier Model**	
1) Classifier is built by the K sub-hyperspheres: when a sample is involved in any sub-hypersphere,it will be seen as positive sample, or negative.	

### Datasets

**Table 1:** LIBSVM datasets.

Dataset name	Feature	Training set	Test set
Adult	119	968	637
madelon	500	1300	700
protein	357	8100	6600

Three datasets from the LIBSVM database^46^ were selected to test the performance of SAE-SVDD. They are Adult, Madelon and protein datasets, which are imbalanced and high-dimensional like the antenatal depression dataset. These datasets were processed to sparse encoding, which was suitable for SAE-SVDD. Then, each dataset was divided into the training set and test set. One class with more samples was selected as the target class, and the other classes were abnormal. More specifically, 50% of the target samples were randomly chosen as the training set; 30% of the target samples and 30% of the abnormal samples were randomly selected as the test set. The description of these data sets is shown in Table 1.

### Metric

Table 2 shows all the possible situations when predicting a sample with a single-class classifier.

**Table 2:** Classification results of single classification.

True class	Prediction	
	+	−
+	TP	FN
−	FP	TN

The target samples are predicted correctly and incorrectly as True Positive (TP) and False Negative (FN). The abnormal samples are predicted correctly and incorrectly as True Negative (TN) and False Positive (FP). In this paper, the *F* score is used as the main metric criterion, a tradeoff metric of precision and recall. The definition of precision *P* and recall *R* is as Eqs. (13) and (14).

13 $$\begin{aligned}{} & {} P = {TP}/{(TP+FP)} \end{aligned}$$

14 $$\begin{aligned}{} & {} R={TP}/{(TP+FN)} \end{aligned}$$

Precision *P* represents the proportion of true target samples in the samples predicted as target classes. The recall *R* represents the proportion of the target samples that are correctly predicted. High recall means that the classifier rarely misreports the target class as an abnormal class. High precision indicates that the abnormal class is rarely misclassified into the target class. The performance of one classifier is usually determined by the recall and precision. However, these two metrics are often in conflict with each other. Then, the *F* score is defined to achieve a balance between recall and precision. And it is shown as Eq. (15).

15 $$\begin{aligned} F={2RP}/{(R+P)} \end{aligned}$$

### Experimental results on three datasets

The SVDD, SA-SVDD, CNN, LSTM, LSTM-Capsule, LSTM-Attention and SAE-SVDD were conducted on the three LIBSVM data sets to compare the performance. SAE-SVDD employed Stacked Auto Encoder to reduce dimension, and the remaining process was the same as SA-SVDD. According to the operation of SAE, the dimension reduction process was conducted layer by layer. Hence, the structure of SAE could be described as the dimension reduction process in Table 3. And all the test results in Table 3 were the average values obtained after ten times of 5-fold cross validation.

**Table 3:** Comparison of classification algorithms.

Algorithm	Adult			Madelon			Protein		
	Dim	*F* score (%)	Runtime (s)	Dim	*F* score (%)	Runtime (s)	Dim	*F* score (%)	Runtime (s)
SVDD	119	83.23	503.72	500	70.43	3302.14	357	58.81	1428.04
SA-SVDD	119	84.12	204.26	500	70.68	3086.93	357	59.26	697.93
CNN	119	72.51	2.31	500	54.56	98.09	357	66.52	418.05
LSTM	119	43.16	3.52	500	40.27	273	357	73.98	1909
LSTM-Capsule	119	75.99	5.72	500	44.67	413.55	357	67.11	2679.38
LSTM-Attention	119	43.27	3.69	500	57.04	221.07	357	**68.17**	1095.49
SAE-SVDD	80	84.52	195.7	400	72.31	718.26	250	60.11	325.61
	60	**86.21**	150.19	300	**72.93**	121.39	100	60.15	105.35
	10	86.10	**94.7**	100	71.52	**67.49**	10	58.98	**46.56**

Best metric values are given in bold.

**Figure 3:**
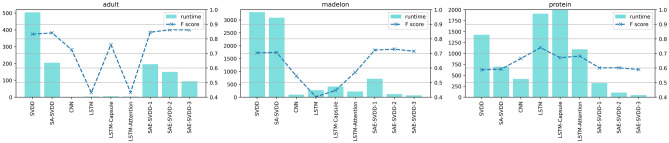
Runtime of classification algorithms on three data sets (SAE-SVDD-1, SAE-SVDD-2 and SAE-SVDD-3 reduce dimension gradually).

It could be seen from Table 3 that the performance of the SA-SVDD was improved after dimension reduction. From Fig. 3, the running time was reduced dramatically, and the *F* score did not change so much. Therefore, SAE-SVDD improved the efficiency of SA-SVDD while it preserved the high performance of SA-SVDD. It also had better performance than four deep learning-based models. The validity of SAE-SVDD to classify imbalanced and high-dimensional datasets could be proved by its good results on the three datasets.

## Experiments on antenatal depression dataset questionnaire

### Data description

The new questionnaire consists of 41 questions, and the questions can be divided into four parts, including natural situation, history of pregnancy and disease, state of mind and demand survey. The questionnaire of the survey is shown in Online Appendix 1. In addition, an antenatal depression and anxiety survey have been conducted in the Jilin Women and Children Hospital^47^. A pregnant woman could finish this questionnaire in few minutes. Finally, 5666 pregnant women participated in the survey, 5259 pregnant women were healthy, and 407 had antenatal depression or anxiety. To build the classifier, the samples needed to be labeled. During the survey, the Self-rating Depression Scale and the Self-assessment Anxiety Scale (SAS)^48,49^ were used to assess the psychological state of pregnant women.

The SDS is a tool for measuring depression. It was designed by William Zung of Duke University in 1965. It was recommended for use by the US Department of Education, Health and Welfare as the scales of psychopharmacology study. It is simple and easy to use, and it can intuitively reflect the subjective feelings of patients’ depression and their changes during treatment. Thus, it has been widely used in rough screening, emotional state assessment, outpatients’ investigation and scientific research. Zung developed the SAS in 1971, and it is a relatively simple clinical tool for analyzing subjective symptoms of patients.In addition, it is believed that SAS can better reflect the personal feelings of psychotic patients with anxiety tendencies. Anxiety is a common emotional disorder in psychological counseling clinics. Therefore, SAS is a popular self-evaluation tool for understanding anxiety symptoms in consultation clinics in recent years.

### Data pre-process

Before running SAE-SVDD, the collected samples were pre-processed as following.

**Table:** 

Workflow of data pre-process	
1.**Sample label:** After each pregnant woman fills out the antenatal depression questionnaire, SDS and SAS are used to assess the psychological state. Then, the target class is defined as normal samples, while abnormal class is defined by depressed or anxious sample	
2.**Denoising:** During the survey, there are a few irrelevant features (such as name, ID), which needs to be cleaned up	
3.**Binarization:** All the feature in the questionnaire values should be binarized, and each sample is described sparsely	
4.**Random sampling:** All the samples are randomly divided into two parts, the training set (80% target class samples) and the test set (20% target class samples and 100% abnormal class samples)	

### Experiment results on antenatal depression dataset

Firstly, the antenatal depression dataset was pre-processed according to the above workflow. Then, in the binarization process, if an option was selected, ‘1’ would be used to represent this option. If the choice was not selected, ‘0’ would be used to represent this option. Finally, we got a vector consisting of ‘0’ and ‘1’, representing the questionnaire. The data obtained by each processing step is shown in Table 4.

**Table 4:** Pre-processing result of antenatal depression dataset.

Raw data	Sample size: 5666
	Characteristic value: 150
Sample label	Target data: 5259
	Abnormal data: 407
	Characteristic value: 150
Denoising, Binarization	Target data: 5259
	Abnormal data: 407
	Characteristic value: 147
Random sampling	Training sample: 4200
	Test sample: 1400
	Characteristic value: 147
Dimensionality reduction	Hidden layer 1: 110
	Hidden layer 2: 90
	Hidden layer 3: 30

### Significance of SAE-SVDD

After the pre-processing was completed, the SAE was applied to the dataset. First, the SAE with three AutoEncoders was constructed, and the neurons numbers of each hidden layer were 110, 90, and 30, respectively. Then, SVDD, SA-SVDD and SAE-SVDD were run ten times with 5-fold cross validation. The average results are shown in Table 5.

**Table 5:** Comparison of classification algorithms on antenatal depression dataset.

Algorithm	Dim	*F* score (%)	Runtime (s)
SVDD	147	85.26	3087.11
SA-SVDD	147	86.65	2654.4
CNN	147	56.60	61.99
LSTM	147	48.14	172.84
LSTM-Capsule	147	54.47	347.29
LSTM-Attention	147	48.14	132.93
SAE-SVDD	110	87.87	2098.24
	90	**87.96**	1504.74
	30	87.63	**431.68**

Best metric values are given in bold.

### The top10 options related to depression selected by the improved TF-IDF

We tried to discover the prevalence and determinants of antenatal depression. A numerical statistic for each questionnaire option was proposed, referring to the term frequency–inverse document frequency (TF-IDF)^50^. TF-IDF is a statistical method to assess the importance of a word for a document in a corpus. If a word appears in a document with a high frequency and rarely appears in other articles, it is considered essential and having a good distinguishing ability for classification. TF-IDF is TF * IDF. TF-IDF of word t in document d from corpus D is shown in Formula (16).

16 $$\begin{aligned}{} & {} TFIDF(t,d,D)=\frac{f_{t,d}}{\Sigma _{t' \in d}t',d} \times \log {\frac{N}{1+n_t}} \end{aligned}$$

17 $$\begin{aligned}{} & {} imp-TFIDF(t)=\frac{p_t}{N_p}\times \log {\frac{N}{1+n_t}} \end{aligned}$$

where the left part represents the word *t*’s frequency, *N* is the size of the corpus, and $n_t$ is the number of documents where the word *t* appears. In this paper, the improved TF-IDF of feature t is defined in Formula (17). The $p_t$ is the number of options *t* selected by pregnant women suffering from antenatal depression or anxiety. $N_p$ is the number of patients, *N* is the number of samples, and $n_t$ is the number of option *t* selected by normal pregnant women. The improved TF-IDF considers the selected options’ distributions in different classes. It is easy to discover the crucial options for the prevalence and determinants of antenatal depression. The top 10 of the most essential options are list in Table 6.

Also, the information gains (IG) of the options were calculated. In IG, the criterion is how much information the feature can bring to the classification system. The more information it gets, the more critical the feature is. For one specific feature, the amount of information will change when the system has it or not. The difference between the amount of information before and after is the feature’s amount of information to the system. The amount of information is entropy. Suppose that the proportion of the $k^{th}$ sample in the current sample set *D* is $p_k (k = 1,2,\ldots , |y|)$. Then the information entropy of D is defined as for formula (18).

18 $$\begin{aligned} Ent(D)=-\sum \nolimits _{k=1}^{|y|}p_k\log _2p_k \end{aligned}$$

Suppose that the discrete attribute *a* has *V* possible values$\{a^1, a^2,\ldots , a^V\}$. If *a* is used to divide the sample set *D*, then *V* branch nodes will be generated. The *v* node contains all the samples in *D* whose value is $a^v$ on attribute *a*, recorded as $D^v$. We can calculate the information entropy of $D^v$ according to equation 18, and then, considering that different branch nodes contain different samples, we can give weight $|D^v|/|D|$ to the branch nodes. The more the number of samples, the greater the influence of the branch nodes, so we can calculate the information gain obtained by dividing the sample set *D* by attribute *a* in Formula (19).

19 $$\begin{aligned} Gain(D,a)=Ent(D)-\sum \nolimits _{v=1}^V\frac{|D^v|}{|D|}Ent(D^v) \end{aligned}$$

The improved TF-IDF considers the selected options’ distributions in different classes. Therefore, it is easy to discover the crucial options that might be the prevalence and determinants of antenatal depression. The top 10 of the most important options are list in Table 6. Generally speaking, the greater the information gain, the more critical it is for classification to use attribute *a*. The essential options’ information gain is also shown in Table 6. Their information gain is significantly higher than the average value, which indicates that the influence factors selected by TF-IDF are practical.

**Table 6:** Top 10 important options by the improved TF-IDF.

Option description	Depression	Normal	Imp-TF-IDF	IG
1. Feel pressure now	138	646	0.3195	0.0044
2. Children will influence the relationship with lover	83	322	0.2537	0.0032
3. Staff	102	578	0.2483	0.0022
4. Occasionally passive smoking	124	937	0.2380	0.0014
5. Own wishes (the reason for wanting a baby)	82	494	0.2133	0.0015
6. Worried about economic pressure	92	707	0.2042	0.0009
7. Cesarean section	73	431	0.2005	0.0014
8. Drinking occasionally	72	443	0.1956	0.0012
9. Passive smoking > 3 hours/day	41	79	0.1864	0.0029
10. General (relationship with colleagues)	44	115	0.1826	0.0025
Average value of all options			0.1005	0.0005

## Discussion

The questionnaire data is encoding sparsely, and the distribution is uneven and loose. The common classifier boundary is difficult to describe. In the proposed SAE-SVDD, the Stack Autoencoder was used to embed the data to a lower dimension. The current data was an equivalent representation of the original data. The running speed of the classier was effectively improved due to the dimension reduction. When it was applied to classify the antenatal depression dataset obtained from the questionnaire, SAE-SVDD discovered pregnant women with antenatal depression effectively and efficiently.

Then, the improved TF-IDF analysis identified important factors of antenatal depression. Through the analysis, stress had a significant impact on antenatal depression in pregnant women, including work stress, psychological stress and financial burden. What’s more, the selected options are much higher than the average.The papers about prenatal depression published recently were reviewed. The reasons mentioned in the papers and the results we got are mutually confirmed. For example, pregnant women in coastal South India faced the same situation, and the possible risk factors were pressure to have a male child and financial difficulties^50^. In Soweto, South Africa, partner and family relationship stressors were central^51^. In rural Maharashtra, a state in India’s western part, feeling pressured to deliver a male child was strongly associated with antenatal depression^52^. Also, in other studies among Chinese women, significant risk factors included financial worries and pregnancy pressure^53^. Besides, pregnant women’s marital status could also affect antenatal depression, including the possible impact of children on the relationship between partners and, lack of support from husbands. In Addis Ababa, Ethiopia, experiencing a shortage of baby’s father support was one of the factors independently associated with antenatal depression^54^. In coastal South India, marital conflict was also one of the possible risk factors^50^. The health status and living environment of pregnant women were also related to antenatal depression, including passive smoking, occasional drinking and cesarean section history.

Compared with the study of pregnant women’s antenatal depression in different countries, some of the factors affecting antenatal depression in Chinese pregnant women are the same as other countries, such as marital status and the relationship between partner, economic pressure, etc. Still, some of the factors are different, such as the living environment of pregnant women.

There are still some limitations in our method, the time cost is quite high, and the encoder can be replaced by some latest methods. We will deal with these problems in future work.

## Conclusion

Antenatal depression is a critical threat to the physical and mental health of pregnant women. The early detection of prenatal depression affects the treatment effect of patients. A questionnaire system was designed in this paper to detect pregnant women’s antenatal depression. The system involved a questionnaire and an analysis model to distinguish patients and normal women. The model performed well on several benchmarks and reached an F score of 86.65% on our questionnaire data. Then we used the information gains and the TF-IDF to find out the precipitating factors of postpartum depression, which are validated by published literature. The new questionnaire analysis model does not only work for antenatal depression discovery in pregnant women. However, it is a general method, and it can be applied to analyze other questionnaires.

## Experiment statement

The questionnaire data in this paper were obtained from the National Free Pregnancy Physical Examinations in Jilin Province, China, in 2014, and the related research results were published in China Practical Medicine in 2017 (Lang, X., Wang, N. Z., Zang, X. & Li, J. A survey of the psychological status of women of planned pregnancy and childbearing age before pregnancy and their needs for counseling and guidance for eugenics. China Practical Medicine 12, 183–185).

We promise that:

1. We have carefully read the Declaration of Helsinki and the experimental procedure is conformed to the provisions of the Declaration.
2. The experiment was approved by Jilin Provincial Institute of Population Science and Technology Ethics Committee.
3. We have obtained informed consent from all subjects for the use of the questionnaire results for research studies.

## Supplementary Information

Supplementary Information 1.

Supplementary Information 2.

## Data Availability

All data generated or analyzed during this study are included in this published article and its supplementary information files.
