# Supplementary material for: A comprehensive psychological tendency prediction model for pregnant women based on questionnaires
Source: Sci Rep. 2023 Jan 2;13:2. doi: 10.1038/s41598-022-26977-3 (PMC9807629; doi:10.1038/s41598-022-26977-3)
Supplement: Supplementary file 1 — Supplementary Information 1. [file 41598_2022_26977_MOESM1_ESM.pdf]

A Questionnaire for Antenatal Depression in Pregnant Women

A.1 The natural situation

0.Number:
1.Name:
2.Age:
3.Ethnicity:
A.ethnic minorities B.Han nationality
4. Occupation:
A.worker B.farmer C.cadre D.medical staff E.Teacher F.staff G.waiter H.business I. no job J.other
5.Education level:
A.master's degree and above B.undergraduate C.specialist D.high school and below
6.Are you smoking?
A.no B.occasionally C.often D.<10 sticks/day E.10-30 sticks/day F.>30 sticks/day
7.Are you passive smoking?
A.no B.occasionally C.often D.<3 hours/day E.>3 hours/day
8.Are you exposed to drugs?
A.no B.yes
9.Are you drinking?
A.no B.occasionally C.liquor D.beer E.wine
10.Are you exposed to toxic and hazardous substances?
A.no B.radiation C.high temperature D.noise E.lead F.mercury G.pesticide H.decoration I.pet J.other
11.What is your family income?
A.2,000 yuan or less B.2000-5000 yuan C.5000-10000 yuan D.10,000 yuan or more

A.2 Pregnancy and disease history

12.Your pregnancy history
A.no B.normal childbirth C.cesarean section D.induced abortion E.spontaneous abortion F.ectopic pregnancy G.premature birth H.stillbirth I.birth defects J.other
13.Do you have or have suffered from the following diseases?
A.no B.heart disease C.chronic nephritis D.high blood pressure E.diabetes F.thyroid disease H.other
14.Do you have or have suffered from the following infectious diseases?
A.no B.tuberculosis C.disease hepatitis D.rubella E.cytomegalovirus F.sexually transmitted diseases G.other:
15.Do you have or have suffered from mental illness?
A.yes B.no C.not sure
16.Are you currently taking the medicine?
A.no B.yes
17.Are you married to a close relative?
A.yes B.no
18.Is there a genetic disease in your family?
A.no B.hemophilia C.congenital heart disease and diabetes D.congenital mental retardation E.blind F.deaf G.dumb H.mental illness F. other J. uncertain

A.3 The state of mind

19.Why do you want a baby?
A.my own wishes B.the wishes of the lover C.the common wishes of husband and wife D.the wishes of parents E.the pressure from other families and friends F.other:
20.What preparations are needed before pregnancy?
A.body B.economy C.psychology D.birth knowledge E.other
21.Are you ready to be a parent?
A.yes B.no C.uncertain
22.Do you think that children will influence the relationship between you and your lover?
A.yes B.no C.not sure
23.Do you care about the child's gender?

A.yes B.no C.uncertain
24.What do you worry about pregnancy?
A.whether the baby is healthy B.economic pressure C.discomfort caused by pregnancy D.labor pain E.baby affects my work and development F.baby occupies my private space G.body shape change H.other:
25.Do you think tension will affect conception?
A.yes B.no C.uncertain
26.Do you think nervous anxiety is caused by miscarriage?
A.yes B.no C.uncertain
27.Do you think mental stimulation affects fetal development?
A.yes B.no C.uncertain
28.Do you think tension can lead to ectopic pregnancy?
A.yes B.no C.uncertain
29.Do you feel pressure now?
A.yes B.no C.uncertain
30.How is your relationship with your lover?
A.harmony B.general C.nervous
31.How is your relationship with friends and relatives?
A.harmony B.general C.nervous
32.How is your relationship with your colleagues?
A.harmony B.general C.nervous
33.Do you currently have a tendency to depression?
A.yes B.no
34.Do you currently have an anxiety tendency?
A.yes B.no

A.4 Demand survey

35.Do you understand the psychological counseling and guidance of pre-pregnancy eugenics?
A.yes B.no
36.Have you received eugenic counseling and guidance before pregnancy?
A.yes B.no
37.Do you think it is necessary to have pre-pregnancy eugenics counseling and guidance?
A.yes B.no C.does not matter
38.How do you know about pre-pregnancy eugenics counseling and guidance?
A.wall newspaper column B.text and publicity materials C.network D.radio and television E.newspapers and magazines F.training lectures G.listen to people around you H.other:
39.Where do you receive pre-pregnancy eugenics counseling and guidance?
A.family planning service station B.maternal and child health center C.general hospital D.professional psychological counseling institutions
40.How did you conduct pre-pregnancy eugenics counseling and guidance?
A.separate consultation B.face-to-face consultation C.telephone consultation D.group training
41.What is your need for counseling and guidance for pre-pregnancy eugenics?

B Psychological questionnaire for antenatal women

Data item	Quantity
Han nationality	5328
minority	338
worker	542
farmer	2380
cadre	80
medical staff	122
teacher	275
staff	651
waiter	255
business	298
unemployed	650
master degree and above	122
bachelor	811
specialist	942
high school and below	3502
do not smoke	5121
occasional smoking	164
smoking<10 sticks / day	30
smoking 10-30 / day	17
smoking>30/day	2
no passive smoking	4079
occasionally passive smoking	989
passive smoking< 3hours / day	82
passive smoking>3 hours/day	120

Data item	Quantity
contact with drugs	34
no drinking	4852
drinking occasionally	477
liquor	11
beer	11
wine	1
no exposure to toxic and hazardous substances	5161
radiation	13
high temperature	13
noise	26
lead	4
mercury	1
pesticide	5
decoration	52
pet	90
family income below 2,000 yuan	1406
family income 2000-5000 yuan	2891
family income 5000-10000 yuan	858
family income more than 10,000 yuan	224
no history of pregnancy	3082
normal childbirth	1060
cesarean section	480
artificial abortion	870
natural abortion	179
ectopic pregnancy	24
premature birth	4
stillborn stillbirth	29
birth defect	9
No heart disease, chronic nephritis, high blood pressure, diabetes, thyroid disease	5238
have or have suffered heart disease	42
have or have suffered chronic nephritis	26
have or have suffered high blood pressure	16
have or have suffered diabetes	9
have or have suffered thyroid disease	32
have not had tuberculosis, viral hepatitis,	

Data item	Quantity
rubella, cytomegalovirus, sexually transmitted diseases	5226
have or have suffered tuberculosis	26
have or have suffered viral hepatitis	66
have or have suffered rubella	21
have or have suffered cytomegalovirus	9
have or have suffered a sexually transmitted disease	11
have or have suffered a mental illness	106
have not had or have had a mental disorder	5210
not sure if you have or have suffered from a mental disorder	44
Are you currently taking the medicine?	334
Are you married to a close relative?	66
no genetic diseases in the family (hemophilia, congenital heart disease, diabetes, congenital mental retardation, blindness, paralysis, dumbness, mental illness)	5281
not sure if there is a genetic disease in the family	17
hemophilia in the family	26
family with congenital heart disease diabetes	4
family with congenital mental retardation	1
family with blind	2
family with deaf	2
family with mute	1
family with mental illness	27
your own wishes (the reason for wanting a baby)	573
the desire of your lover (the reason for wanting a baby)	401
the common wishes of husband and wife (the reason for wanting a baby)	4279
the wishes of parents (the reason for wanting a baby)	602
the pressure from other families and friends (the reason for wanting a baby)	29
body(what preparations are needed before pregnancy)	4067
economy(what preparations are needed before pregnancy)	2150
psychology(what preparations are needed before pregnancy)	2296
birth knowledge (what preparations are needed before pregnancy)	2521
be prepared for parents	4730
not ready for parents	422
not sure if you are ready for parents	202
children will influence the relationship with your lover	398
children will not affect the relationship with your lover	4610
not sure whether child will affect the relationship with the lover	345
concerned about the child's gender	538

Data item	Quantity
don’t care about the child’s gender	4582
not sure if you care about your child’s gender	240
worried about whether your baby is healthy	4488
worried about economic pressure	782
worried about the discomfort caused by pregnancy	729
worried about labor pain	601
baby affects work and development	141
worried that the baby occupies your own private space	207
worried about body shape changes	337
think that tension will affect conception	2713
think that tension does not affect conception	1639
not sure whether tension affects conception	1020
think that nervous anxiety can cause miscarriage	3046
think that nervous anxiety and fear will not cause miscarriage	1138
not sure whether nervous anxiety or fear causes abortion	1168
think that mental stimulation affects fetal development	3279
think that mental stimulation does not affect fetal development	1001
not sure whether mental stimulation affects fetal development	1070
think that tension can lead to ectopic pregnancy	2239
think that tension does not lead to ectopic pregnancy	1371
not sure whether tension leads to ectopic pregnancy	1732
feel pressure now	774
don’t feel pressure at the moment	3999
not sure if there is pressure now	585
harmony(the relationship with the lover)	5246
general(relationship with a lover)	83
nervous (relationship with a lover)	26
harmony(with friends and relatives)	5214
general(relationship with relatives and friends)	112
nervous(relationship with relatives and friends)	26
harmony(relationship with colleagues)	5163
general(relationship with colleagues)	155
nervous(relationship with colleagues)	24
is there a tendency to depression	345
is there an anxiety tendency at present	205
do you know about pre-pregnancy eugenics counseling and guidance	2736
have you received eugenics counseling and guidance before pregnancy	2261
pre-pregnancy eugenics counseling and guidance are necessary	3534

Data item	Quantity
there is no need for pre-pregnancy eugenics counseling and guidance	970
no pre-pregnancy eugenics counseling and guidance	818
through the wall newspaper column to understand the psychological counseling and guidance of pre-pregnancy eugenics	855
understanding the psychological counseling and guidance of pre-pregnancy eugenics through texts and propaganda	1052
understanding the psychological counseling and guidance of pre-pregnancy eugenics through the network	2492
understanding the psychological counseling and guidance of pre-pregnancy eugenics through radio and television	1354
through newspapers and magazines to understand the psychological counseling and guidance of pre-pregnancy eugenics	313
through training seminars to understand the psychological counseling and guidance of pre-pregnancy eugenics	704
by listening to people around you to understand the psychological counseling and guidance of pre-pregnancy eugenics	838
family planning service station (accepting pre-pregnancy eugenics counseling and guidance location)	3046
maternal and child health center(receiving pre-pregnancy eugenics counseling and guidance locations)	1402
general hospital (accepting pre-pregnancy eugenics counseling and guidance locations)	864
professional psychological counseling agency(accepting pre-pregnancy eugenics counseling and guidance locations)	539
consultation alone (pre-pregnancy eugenics counseling and guidance)	1227
face-to-face consultation (pre-pregnancy eugenics counseling and guidance)	1954
telephone consultation (pre-pregnancy eugenics counseling and guidance)	1142
group training(pre-pregnancy eugenics counseling and guidance)	1025
not sure if you care about your child's gender	240
worried about whether your baby is healthy	4488
worried about economic pressure	782
worried about the discomfort caused by pregnancy	729
worried about labor pain	601
baby affects work and development	141

Data item	Quantity
worried that the baby occupies your own private space	207
worried about body shape changes	337
think that tension will affect conception	2713
think that tension does not affect conception	1639
not sure whether tension affects conception	1020
think that nervous anxiety can cause miscarriage	3046
think that nervous anxiety and fear will not cause miscarriage	1138
not sure whether nervous anxiety or fear causes abortion	1168
think that mental stimulation affects fetal development	3279
think that mental stimulation does not affect fetal development	1001
not sure whether mental stimulation affects fetal development	1070
think that tension can lead to ectopic pregnancy	2239
think that tension does not lead to ectopic pregnancy	1371
not sure whether tension leads to ectopic pregnancy	1732
feel pressure now	774
don't feel pressure at the moment	3999
not sure if there is pressure now	585
harmony(the relationship with the lover)	5246
general(relationship with a lover)	83
nervous (relationship with a lover)	26
harmony(with friends and relatives)	5214
general(relationship with relatives and friends)	112
nervous(relationship with relatives and friends)	26
harmony(relationship with colleagues)	5163
general(relationship with colleagues)	155
nervous(relationship with colleagues)	24
is there a tendency to depression	345
is there an anxiety tendency at present	205
do you know about pre-pregnancy eugenics counseling and guidance	2736
have you received eugenics counseling and guidance before pregnancy	2261
pre-pregnancy eugenics counseling and guidance are necessary	3534
there is no need for pre-pregnancy eugenics counseling and guidance	970
no pre-pregnancy eugenics counseling and guidance	818
through the wall newspaper column to understand the psychological counseling and guidance of pre-pregnancy eugenics	855
understanding the psychological counseling and guidance of pre-pregnancy eugenics through texts and propaganda	1052

Data item	Quantity
understanding the psychological counseling and guidance of pre-pregnancy eugenics through the network	2492
understanding the psychological counseling and guidance of pre-pregnancy eugenics through radio and television	1354
through newspapers and magazines to understand the psychological counseling and guidance of pre-pregnancy eugenics	313
through training seminars to understand the psychological counseling and guidance of pre-pregnancy eugenics	704
by listening to people around you to understand the psychological counseling and guidance of pre-pregnancy eugenics	838
family planning service station (accepting pre-pregnancy eugenics counseling and guidance location)	3046
maternal and child health center(receiving pre-pregnancy eugenics counseling and guidance locations)	1402
general hospital (accepting pre-pregnancy eugenics counseling and guidance locations)	864
professional psychological counseling agency(accepting pre-pregnancy eugenics counseling and guidance locations)	539
consultation alone (pre-pregnancy eugenics counseling and guidance)	1227
face-to-face consultation (pre-pregnancy eugenics counseling and guidance)	1954
telephone consultation (pre-pregnancy eugenics counseling and guidance)	1142
group training(pre-pregnancy eugenics counseling and guidance)	1025
